# Between the uniform and the toll: burnout and mental health among Brazilian Military Police

**DOI:** 10.1590/0034-7167-2025-0429

**Published:** 2026-09-28

**Authors:** Caroline Schacker Evangelista, Kaihara de Freitas Furtado, Vitória Silva da Rosa, Ana Cristina Wesner Viana, Elisabete Maria das Neves Borges, Adriana Aparecida Paz

**Affiliations:** I Universidade Federal de Ciências da Saúde de Porto Alegre. Porto Alegre, Rio Grande do Sul, Brazil; II Hospital de Clínicas de Porto Alegre. Porto Alegre, Rio Grande do Sul, Brazil; III Escola Superior de Enfermagem do Porto. Porto, Portugal

**Keywords:** Burnout, Occupational Stress, Police, Technology, Health, Occupational Health., Burnout, Estrés Laboral, Policía, Tecnología para la Salud, Salud Laboral.

## Abstract

**Objectives:**

to examine the association between burnout and sociodemographic, occupational, and health-related factors among Brazilian Military Police officers.

**Methods:**

cross-sectional study conducted between November and December 2024 with 1,028 participants. Burnout was assessed using the Burnout Assessment Tool, with descriptive and inferential analyses.

**Results:**

most participants were men (81.9%) and Brown (pardo) (49.6%); mean age was 41.9 ± 6.5 years. Burnout ranged from moderate to very high in 75.4%. Age, years of service, and physical inactivity were negatively correlated with burnout. Scores were significantly higher among women, White participants, those with a college degree, fixed shifts, physical inactivity, long-term medication use, prior psychological care, and addiction.

**Conclusions:**

burnout reflects an imbalance between high job demands and limited institutional resources, consistent with the Job Demands-Resources model. Findings underscore the need for interdisciplinary policies, healthy workplaces, and mental health care aligned with SDGs 3 and 8, reinforcing nursing’s strategic role.

## RESUMEN

**Objetivos:**

analizar la asociación entre *burnout* y factores sociodemográficos, laborales y de salud en policías militares brasileños.

**Métodos:**

estudio transversal realizado entre noviembre y diciembre de 2024 con 1.028 participantes. El *burnout* se evaluó mediante el *Burnout Assessment Tool*, con análisis descriptivos e inferenciales.

**Resultados:**

en el 75,4% de los participantes, el *burnout* osciló entre moderado y muy alto. La edad, los años de servicio y la inactividad física se correlacionaron negativamente con el *burnout*. Se observaron medias significativamente más altas en mujeres, personas blancas, participantes con educación superior, turno fijo, inactividad física, uso continuado de medicación, antecedentes de atención psicológica y adicción.

**Conclusiones:**

los hallazgos evidencian un desequilibrio entre demandas laborales y escasez de recursos institucionales, en consonancia con el modelo de demandas y recursos laborales. Urgen políticas interdisciplinarias, entornos saludables y atención a la salud mental, alineadas con los ODS 3 y 8, con protagonismo de la enfermería.

## INTRODUCTION

Work-related mental disorders are currently among the leading causes of illness among professionals in complex organizational settings, posing major challenges to public health and occupational health and safety in the 21st century^(1)^. Burnout stands out among them and is characterized by exhaustion, mental distance, and impaired cognitive and emotional functioning resulting from chronic exposure to occupational stress^(2)^.

Since its inclusion in the International Classification of Diseases (ICD-11) by the World Health Organization (WHO) in 2022, burnout has been officially recognized as an occupational phenomenon, underscoring the need for institutional strategies focused on workplace mental health surveillance, prevention, and care^(3)^. Despite these conceptual advances, applied studies examining burnout in occupations highly exposed to psychosocial risks, such as public safety, remain scarce.

Brazilian Military Police (Polícia Militar, PM) - a state-level uniformed patrol force responsible for maintaining public order - represents one of the occupational groups most vulnerable to psychosocial strain. PMs are daily exposed to adverse conditions such as irregular work schedules, frequent violence, resource shortages, and organizational tensions, which affect not only individual well-being but also institutional performance and, consequently, public safety^(4-6)^. Illness among PMs also affects social stability, the quality of public services, and public trust in public safety institutions^(7)^.

To understand this scenario, theoretical frameworks are needed that can account for the multiple dimensions of work. In this regard, the Job Demands-Resources (JD-R) model, proposed by Demerouti et al.^(8)^ and later expanded by Bakker and Demerouti^(9)^, is particularly useful. According to this model, burnout arises from an imbalance between job demands (emotional, physical, and cognitive) and the resources available to cope with them (e.g., institutional support, autonomy, and recognition)^(10)^. This approach allows an integrated analysis of the interplay between the individual and organizational factors involved in occupational distress.

Based on this framework, the Burnout Assessment Tool (BAT) was developed as a more comprehensive alternative to the classic Maslach Burnout Inventory (MBI). The BAT organizes burnout into four core dimensions (Exhaustion, Mental Distance, Cognitive Impairment, and Emotional Impairment) and two secondary dimensions (Psychological complaints and Psychosomatic complaints), providing a more integrated understanding of work’s psychosocial effects on individuals^(2,11)^. In addition, by incorporating Mental Distance, Cognitive Impairment, and Emotional Impairment as components of burnout, the BAT reflects a contemporary theoretical framework and addresses the limitations of the MBI^(12)^.

In this study, we used the BAT-33, work-related version, because it is well suited to the occupational context and can assess symptoms linked to job demands and working conditions^(2,11)^. This choice also reflects the need to generate evidence to inform public and institutional policies, recognizing mental health as an inseparable component of occupational health and strengthening prevention, comprehensive care, and recognition of PMs’ work. A key strength of this study is the use of the BAT-33, a more comprehensive and theoretically consistent instrument than the traditional MBI because it encompasses cognitive and emotional dimensions that remain underexplored in the Brazilian literature. By identifying vulnerable groups and factors associated with burnout among Brazilian PMs, this study broadens the scientific debate and makes a novel contribution to the development of evidence-based strategies to address burnout.

## OBJECTIVES

To analyze the association between burnout and sociodemographic, occupational, and health related factors among Brazilian Military Police officers.

## METHODS

### Ethical aspects

The study was conducted in accordance with national and international ethical guidelines and was approved by the Research Ethics Committee of the Federal University of Health Sciences of Porto Alegre (UFCSPA). The study fully complied with Brazilian National Health Council Resolution No. 466/2012^(13)^, which governs research involving human participants. It also followed specific recommendations for online research, as well as the privacy and personal data protection principles outlined in Law No. 13,709/2018 (Brazil’s General Data Protection Law [LGPD])^(14)^.

### Study design, period, and setting

This was a cross-sectional study with a quantitative, descriptive, and analytical approach. The study was reported in accordance with the Strengthening the reporting of observational studies in epidemiology (STROBE) guidelines. It was conducted through the online scientific outreach platforms of the Research Group on Technology, Management, Education, and Occupational Safety (TeGEST), affiliated with UFCSPA and registered in the CNPq Directory of Research Groups (DGP CNPq). Recruitment was carried out through the group’s Facebook^®^ page, active since January 2019, and its official Instagram^®^ profile. The invitation was posted with the group deputy leader’s written authorization.

### Population and inclusion and exclusion criteria

The study population consisted of 1,574 Brazilian PMs. The sample was recruited by convenience sampling using snowball sampling, beginning with invitations posted on TeGEST’s social media accounts and encouraging participants to share them with colleagues in the profession. In addition, electronic messages were sent to Public Security Secretariats, General Commands, Casas Militares, and associations representing officers and enlisted personnel in all Brazilian states, requesting support in disseminating the study among personnel in the state Military Police forces. Participants were eligible if they were career PMs, were on active duty, and held permanent employment status. Retired PMs, those on temporary leave for more than 30 consecutive days (for reasons such as sick leave, vacation, or similar leave), temporarily hired personnel, and those still in their probationary period were excluded. After the eligibility criteria were applied and response completeness was verified, the final sample consisted of 1,028 PMs from multiple Brazilian states.

### Study protocol

The questionnaire was made available online (https://bit.ly/PMQuestBR) and via QR code. The form included the eligibility criteria and the Informed Consent Record (*Registro de Consentimento Livre e Esclarecido* [RCLE]), with an option to download the full document. Participation was confirmed through electronic consent recorded in the form.

Data were collected between November and December 2024 using an online questionnaire with two sections: (1) Burnout, assessed with the BAT-33, work-related version; and (2) PMs’ sociodemographic, occupational, and health characteristics. The BAT was translated and adapted for use in Brazil, with evidence of validity and reliability reported in a Brazilian sample of workers (n = 2,217) from multiple occupational groups as part of a cross-cultural study that also included Portuguese workers (n = 886). The instrument comprises 33 items across four core dimensions (Exhaustion, Mental distance, Cognitive impairment, and Emotional impairment) and two secondary dimensions (Psychological complaints and Psychosomatic complaints)^(2)^. The work-related version was selected because it is well suited to the occupational context and allows assessment of how job demands and working conditions affect mental health. Its use has also been recommended for occupational groups exposed to high demands and stress, which supports its applicability to the population under study^(2,15)^.

### Data analysis and statistics

Data were stored in Google Drive^®^ as a Google Sheets^®^ spreadsheet and exported as a Microsoft Excel^®^ file to a local computer, ensuring anonymity and protection of sensitive data. Variables were coded according to a study-specific coding manual to ensure consistency and standardize selected responses. The dataset was then imported into Statistical Package for the Social Sciences® for descriptive and inferential analyses. Variables were summarized using absolute and relative frequencies, measures of central tendency and dispersion, and percentiles, depending on the data type and distribution. Normality was assessed with the Kolmogorov-Smirnov test (p < 0.05). Pearson’s correlation coefficient and the independent-samples *t* test were used for inferential analyses, with statistical significance set at 5%.

## RESULTS

The study included 1,028 PMs from 225 municipalities across 20 Brazilian federal units. The North Region accounted for the largest share of participants (491; 47.8%), followed by the South Region (284; 27.6%), Central-West Region (134; 13.0%), Northeast Region (111; 10.8%), and Southeast Region (7; 0.7%); one participant did not report their region of origin (1; 0.1%). Most participants were men (842; 81.9%) and heterosexual (996; 96.9%), with a mean age of 41.9 ± 6.5 years (range: 23 to 60 years). Regarding race/ethnicity, participants self-identified as Brown (pardo) (510; 49.6%), White (387; 37.6%), Black (118; 11.5%), Asian (5; 0.5%), and Indigenous (4; 0.4%); 4 (0.4%) did not report this information. Regarding marital status, 807 (78.5%) were married or living with a partner. The predominant educational attainment was a college degree (817; 79.5%), followed by high school (177; 17.2%), technical and vocational education (24; 2.3%), and elementary school (8; 0.8%); 2 (0.2%) did not report this information. Table 1 shows the PMs’ occupational and health characteristics.

**Table 1:** Occupational and health characteristics of Brazilian Military Police, Brazil, 2025

Occupational and health characteristics	n (%)
**Rank**	
Sergeant	438 (42.5)
Corporal	158 (15.4)
Private	127 (12.3)
Lieutenant	85 (8.3)
Major	58 (5.6)
Lieutenant colonel	53 (5.2)
Subtenente (warrant officer)	52 (5.1)
Captain	39 (3.8)
Colonel	15 (1.5)
Did not report	3 (0.3)
**Tempo de atuação (em anos)**	17.6 (7.7)^*^
**Role**	
Administrative duties	450 (43.8)
Patrol duties	416 (40.5)
Administrative and patrol duties	48 (4.7)
Other duties	90 (8.7)
Did not report	24 (2.3)
**Work shift**	
Rotating	492 (47.9)
Morning and afternoon	314 (30.4)
Afternoon	124 (12.1)
Morning	84 (8.2)
Night	12 (1.2)
Did not report	2 (0.2)
**Weekly working hours**	43.8 (9.9)^*^
**Federal unit**	
Pará	307 (29.8)
Santa Catarina	161 (15.7)
Rio Grande do Sul	123 (11.9)
Goiás	109 (10.6)
Bahia	79 (7.7)
Tocantins	74 (7.2)
Rondônia	60 (5.8)
Acre	24 (2.3)
Alagoas	20 (1.9)
Roraima	15 (1.5)
Mato Grosso	13 (1.3)
Amazonas	11 (1.1)
Distrito Federal	8 (0.8)
Rio de Janeiro	6 (0.6)
Sergipe	6 (0.6)
Maranhão	4 (0.4)
Mato Grosso do Sul	4 (0.4)
São Paulo	1 (0.1)
Pernambuco	1 (0.1)
Rio Grande do Norte	1 (0.1)
Não informou	1 (0.1)
**Physical activity (yes)**	849 (82.6)
Physical activity frequency (days)	3.3 (1.4)^*^
**Long-term medication use (yes)**	332 (32.3)
**Psychological care (yes)**	452 (44)
Care received within the corporation	185 (40.9)
Prefer not to say	68 (6.5)
**Any addiction (yes)**	212 (20.6)
Substances used	1.2 (0.5)^*^

* *Mean ± standard deviation.*

Most PMs (860; 83.6%) held lower ranks, including private, corporal, sergeant, *subtenente* (warrant officer), and lieutenant. Years of service ranged from 1 to 40 years, with most PMs having served up to 19 years in the force, and 534 (51.9%) were assigned to fixed shifts. Among the 90 participants classified under “Other roles”, 23 (25.5%) worked in health services; 20 (22.3%) in Casa Militar; 12 (13.3%) in Civil Defense; 9 (10.1%) as intelligence agents; 8 (8.9%) in guard duty; 6 (6.7%) in educational units; 3 (3.3%) in the Emergency Center; 3 (3.3%) in the Cavalry; 2 (2.2%) as armorers; 2 (2.2%) in the Artistic Unit; and 2 (2.2%) in Special Operations. Among participants reporting addiction, only 149 (70.2%) specified the substance used, yielding 192 responses and a mean of 1.2 ± 0.5 substances per respondent. The most commonly reported substances were alcohol (87; 45.3%), followed by tobacco (38; 19.8%), compulsive eating (26; 13.5%), nonprescription medication use (16; 8.3%), and marijuana (1; 0.5%). Of note, 24 (12.6%) participants chose to report other addictions, although marijuana, cocaine, ecstasy, and crack were listed as response options. Another 63 (29.8%) participants who reported having an addiction preferred not to disclose the type of addiction or did not answer this item. Table 2 presents the prevalence of burnout symptoms.

**Table 2:** Prevalence of burnout symptoms among Brazilian Military Police, Brazil, 2025

Dimensions and domains	Mean (SD^*^)	Low n (%)	Moderate n (%)	High n (%)	Very high n (%)	IC 95% Cronbach’s α
**BAT-33**	2.2 (0.7)	253 (24.6)	516 (50.2)	206 (20)	53 (5.2)	1.65-3.14 0.968
**Core symptoms**	2.2 (0.7)	268 (26.1)	506 (49.2)	202 (19.6)	52 (5.1)	1.65-2.90 0.958
Exhaustion	2.6 (0.8)	253 (24.6)	504(49)	228 (22.2)	43 (4.2)	2.22-2.90 0.930
Mental distance	1.9 (0.9)	308 (30)	464 (45.1)	207 (20.1)	49 (4.8)	1.70-2.44 0.873
Cognitive impairment	2.1 (0.8)	267 (26)	517 (50.2)	200 (19.5)	44 (4.3)	1.91-2.54 0.886
Emotional impairment	1.8 (0.7)	329 (32)	450 (43.7)	198 (19.3)	51 (5.0)	1.65-2.18 0.904
**Secondary symptoms**	2.4 (0.8)	292 (28.4)	499 (48.5)	187 (18.2)	50 (4.9)	1.77-3.14 0.921

* *SD - standard deviation.*

Age was weakly, negatively, and significantly correlated with all BAT-33 dimensions and domains, indicating slightly lower burnout scores with increasing age: BAT-33 (r = -0.114; p < 0.001), core symptoms (r = -0.111; p < 0.001), Exhaustion (r = -0.128; p < 0.001), Mental Distance (r = -0.070; p = 0.024), Cognitive Impairment (r = -0.100; p = 0.001), Emotional Impairment (r = -0.071; p = 0.022), and secondary symptoms (r = -0.105; p = 0.001). Years of service as a PM showed a negative correlation with Exhaustion (r = -0.085; p = 0.007), suggesting lower exhaustion among more experienced officers. No significant associations were found between weekly working hours and BAT-33 scores (p > 0.05).

Weekly frequency of physical activity showed negative and significant correlations with all BAT-33 dimensions and domains: BAT-33 (r = -0.149; p < 0.001), core symptoms (r = -0.138; p < 0.001), Exhaustion (r = -0.145; p < 0.001), Mental Distance (r = -0.088; p = 0.009), Cognitive Impairment (r = -0.118; p < 0.001), Emotional Impairment (r = -0.127; p < 0.001), and secondary symptoms (r = -0.151; p < 0.001). By contrast, the number of substances consumed by participants reporting addiction was positively correlated with all BAT-33 dimensions and domains: BAT-33 (r = 0.325; p < 0.001), core symptoms (r = 0.316; p < 0.001), Exhaustion (r = 0.319; p < 0.001), Mental Distance (r = 0.240; p = 0.003), Cognitive Impairment (r = 0.224; p = 0.006), Emotional Impairment (r = 0.305; p < 0.001), and secondary symptoms (r = 0.308; p < 0.001). Table 3 shows the associations between the BAT-33 dimensions and domains and PMs’ sociodemographic, occupational, and health variables.

**Table 3:** Association between burnout and Military Police officers’ sociodemographic, occupational, and health characteristics, Brazil, 2025

Variables	BAT-33		Core symptoms		Exhaustion		Mental distance		Cognitive impairment		Emotional impairment		Secondary symptoms	
	Mean (SD^*^)	*p* value^†^	Mean (SD^*^)	*p* value^†^	Mean (SD^*^)	*p* value^†^	Mean (SD^*^)	*p* value^†^	Mean (SD^*^)	*p* value^†^	Mean (SD^*^)	*p* value^†^	Mean (SD^*^)	*p* value^†^
**Sex**														
Male	2.2(0.7)	**0.008**	2.1(0.7)	0.097	2.6(0.8)	0.238	1.9(0.9)	0.925	2.1(0.8)	**0.001**	1.8(0.7)	0.105	2.3(0.8)	**<0.001**
Female	2.4(0.7)		2.2(0.6)		2.7(0.7)		1.9(0.8)		2.3(0.8)		1.9(0.8)		2.6(0.8)	
**Race/ethnicity**														
Brown. Black, Indigenous, and Asian	2.1(0.7)	**< 0.001**	2.1(0.7)	**< 0.001**	2.5(0.8)	**0.014**	1.8(0.8)	**< 0.001**	2.0(0.8)	**< 0.001**	1.7(0.7)	**< 0.001**	2.3(0.8)	**< 0.001**
White	2.4(0.7)		2.3(0.7)		2.7(.0.8)		2.0(0.9)		2.3(0.8)		1.9(0.8)		2.5(0.8)	
**Educational attainment**														
High school	2.1(0.7)	**< 0.001**	2.0(0.7)	**< 0.001**	2.4(0.8)	**0.002**	1.8(0.8)	0.058	1.9(0.8)	**< 0.001**	1.7(0.7)	**0.002**	2.2(0.8)	**< 0.001**
College degree	2.3(0.7)		2.2(0.7)		2.6(0.8)		1.9(0.9)		2.2(0.8)		1.8(0.8)		2.4(0.8)	
**Rank**														
Lower ranks	2.2(0.7)	0.221	2.2(0.7)	0.226	2.6(0.8)	0.228	2.0(0.9)	**0.002**	2.1(0.9)	0.371	1.8(0.8)	0.535	2.4(0.9)	0.278
Higher ranks	2.2(0.6)		2.1(0.6)		2.5(0.7)		1.8(0.8)		2.1(0.7)		1.8(0.7)		2.3(0.7)	
**Years of service**														
≤ 19 anos	2.2(0.7)	0.277	2.2(0.7)	0.270	2.6(0.8)	**0.014**	1.9(0.9)	0.540	2.1(0.8)	0.833	1.8(0.7)	0.918	2.4(0.8)	0.355
≥ 20 anos	2.2(0.7)		2.1(0.7)		2.5(0.8)		1.9(0.9)		2.1(0.8)		1.8(0.7)		2.3(0.8)	
**Work shift**														
Fixed	2.3(0.7)	0.159	2.2(0.7)	0.264	2.6(0.8)	0.748	1.9(0.8)	0.509	2.2(0.8)	**< 0.001**	1.8(0.7)	0.235	2.4(0.8)	0.070
Rotating	2.2(0.7)		2.1(0.7)		2.6(0.8)		1.9(0.9)		2.0(0.8)		1.8(0.8)		2.3(0.8)	
**Physical activity**														
No	2.4(0.7)	**< 0.001**	2.3(0.7)	**0.001**	2.8(0.8)	**< 0.001**	2.1(0.9)	**0.002**	2.3(0.8)	**0.004**	2.0(0.8)	**0.009**	2.7(0.8)	**< 0.001**
Yes	2.2(0.7)		2.1(0.7)		2.6(0.8)		1.9(0.9)		2.1(0.8)		1.8(0.7)		2.3(0.8)	
**Long-term medication use**														
No	2.1(0.6)	**< 0.001**	2.0(0.6)	**< 0.001**	2.5(0.8)	**< 0.001**	1.8(0.8)	**< 0.001**	2.0(0.7)	**< 0.001**	1.7(0.7)	**< 0.001**	2.2(0.7)	**< 0.001**
Yes	2.5(0.7)		2.4(0.7)		2.8(0.8)		2.1(0.9)		2.4(0.9)		2.0(0.8)		2.7(0.8)	
**Psychological care**														
No	1.9(0.5)	**< 0.001**	1.9(0.5)	**< 0.001**	1.9(0.5)	**< 0.001**	1.6(0.7)	**< 0.001**	1.8(0.7)	**< 0.001**	1.5(0.5)	**< 0.001**	2.0(0.6)	**< 0.001**
Yes	2.6(0.7)		2.5(0.7)		2.5(0.8)		2.2(0.9)		2.5(0.8)		2.1(0.8)		2.8(0.8)	
**Addiction**														
No	2.2(0.7)	**< 0.001**	2.1(0.7)	**< 0.001**	2.5(0.8)	**< 0.001**	1.8(0.8)	**< 0.001**	2.0(0.8)	**< 0.001**	1.7(0.7)	**< 0.001**	2.3(0.8)	**< 0.001**
Yes	2.5(0.8)		2.4(0.8)		2.8(0.8)		2.2(1.0)		2.4(0.9)		2.1(0.9)		2.6(0.9)	

* *Standard deviation; †significance level (p < 0.05).*

The analysis of factors associated with burnout among PMs showed greater vulnerability among women, who had higher mean scores on the BAT-33, Cognitive Impairment, and secondary symptoms. White officers also had higher scores across all BAT-33 dimensions and domains, suggesting possible sociocultural and occupational influences. Officers with a college degree had higher mean scores in most dimensions, except Mental distance, which may reflect greater awareness of or exposure to occupational stressors.

Rank was significantly associated with Mental distance, particularly among lower-ranking personnel (privates, corporals, sergeants, warrant officers, and lieutenants), possibly because of heavier workloads and lower autonomy. Exhaustion was more pronounced among PMs with up to 19 years of service, suggesting greater strain during midcareer stages. Fixed shifts were associated with Cognitive impairment, suggesting that rigid work schedules may negatively affect occupational health.

Regular physical activity was a protective factor, with lower scores across all BAT-33 dimensions and domains. By contrast, long-term medication use was associated with higher mean scores, possibly reflecting comorbidities or less effective coping strategies. Officers receiving psychological care also had higher scores, suggesting higher stress levels. Substance use was associated with greater psychosocial impairment, highlighting risk behaviors linked to burnout. No statistically significant associations were found between the BAT-33 dimensions and domains and the variables “age over 40 years”, “marital status”, and “availability of psychological care within the corporation” (p > 0.05), suggesting that these factors did not directly influence burnout levels.

## DISCUSSION

The findings can be interpreted through the lens of the JD-R model, which conceptualizes burnout as the result of an imbalance between high job demands and limited institutional and personal resources. In policing, these demands include exposure to risk, hierarchical pressure, long working hours, and high emotional demands, whereas resources such as social support, recognition, and autonomy remain limited. The BAT makes the effects of this imbalance visible across measurable dimensions, such as Exhaustion, Mental distance, Cognitive impairment, and Emotional impairment, allowing burnout to be understood as both an individual and a structural phenomenon and underscoring the need for institutional policies that strengthen coping resources and protect PMs’ mental health^(16,17)^.

related effects on mental health and acknowledging the psychosocial complexity of occupational distress^(2,10,18)^. In this regard, cognitive Impairment, manifested in memory lapses and slowed information processing, underscores the importance of preventive and strategic interventions to preserve PMs’ operational capacity^(5,19-22)^. The instrument also reframes cynicism as Mental distance, understood as a process of progressive cognitive and affective detachment associated with a loss of meaning and professional engagement^(2,15,18)^. Finally, by assessing secondary symptoms such as sleep disturbances, tachycardia, headaches, muscle pain, and gastrointestinal discomfort, the instrument captures manifestations of chronic stress that are often normalized in everyday police work^(2,15,18)^.

The sample was predominantly male, with a mean age above 40 years, reflecting an aging workforce within a rigid hierarchical structure. Women appear more vulnerable to burnout, especially in relation to exhaustion, which may be intensified by male-dominated environments that render occupational distress invisible^(5,23,24)^. Age and years of service are also relevant factors, as they reflect cumulative wear and prolonged exposure to stress^(25)^. Most participants serve in lower ranking positions, work rotating shifts, and take on multiple duties, a combination that increases the risk of exhaustion and mental distance^(6,26)^. Dissatisfaction with the hierarchical structure and the overload borne by lower-ranking personnel suggest that illness stems not only from individual factors, but also from the scarcity of coping resources provided by the organizational model.

Having a college degree does not necessarily translate into career advancement, which can lead to frustration and low professional fulfillment^(27)^. Recognition from superiors positively influences mental health, whereas its absence contributes to occupational stress^(4,27-29)^. White PMs had higher burnout scores, possibly because of greater visibility, institutional demands, and self imposed pressure, whereas Black PMs may report less distress because emotional suffering is more often normalized or because they distrust the institution’s ability to preserve confidentiality^(30-32)^.

Severe burnout symptoms are associated with intentions to leave the profession prematurely, work overload, and low organizational support, pointing to a risk to retention^(33)^. A similar study conducted with police officers in Thailand found that work overload, combined with hierarchical rigidity and lack of recognition, has intensified psychological exhaustion and the desire to leave the profession^(34)^. In settings marked by high demands and low subjective return, burnout contributes to illness and demotivation, undermining mental health and retention in the profession^(16,17)^.

Continuous exposure to pain, violence, and vulnerability leads to emotional depletion and affective exhaustion, fostering the pattern of mental distance identified among PMs as a strategy of self-preservation^(35)^. This mental distance, often expressed as indifference or cynicism, does not necessarily indicate contempt but rather a way of coping with chronic stress^(2,11,15,18)^.

Although many PMs reported being in good health, the data also show medication use, psychological care-seeking, and reports of addiction, suggesting a mismatch between perceived health and the reality of psychological distress. In military settings, where emotional toughness is highly valued, acknowledging vulnerability may be seen as weakness, which contributes to institutional denial of illness and delays access to appropriate care^(36)^. Studies show that, in the absence of safe spaces for emotional expression, many PMs resort to individual strategies such as self-medication, substance use, and isolation, which, although palliative, tend to worsen burnout and mental distance^(37,38)^.

However, these findings do not necessarily indicate that institutional care policies are in place. On the contrary, they may point to a lack of structural responses and to the individualization of responsibility for mental health^(39)^. One study found that, despite the high prevalence of mental disorders, most PMs rely on self-directed care without adequate institutional support^(40)^. Barriers such as stigma and distrust of services provided by the corporation itself limit help-seeking, while the accumulation of everyday stressors heightens psychological vulnerability^(41)^. This scenario underscores the need for systematic and ongoing interventions that go beyond individual-level responsibility.

Mental health continues to be treated as an individual responsibility, while institutional responses remain limited, and stigma and distrust within the corporation restrict access to appropriate care and engagement in self-care practices within a culture of invulnerability^(5,35)^. Lack of institutional support may discourage PMs from seeking help, thereby worsening symptoms of stress and exhaustion and increasing the risk of conditions such as post-traumatic stress disorder (PTSD)^(42)^. By contrast, structured mindfulness-based interventions have shown promise in fostering mindfulness, improving emotional regulation, and reducing burnout, especially when implemented in hierarchical settings with organizational support^(43-45)^.

The inclusion of PMs from different regions, with strong representation from the North and South, together with the study’s sample size, adds robustness to the findings and suggests that burnout among PMs is a systemic problem. Recurrent exposure to violence, reduced work ability, and alcohol use were associated with poorer mental health and a higher risk of burnout, confirming convergent findings in the Brazilian literature, which identifies work overload and risk behaviors as central elements in illness among police personnel^(36,46)^. Perceived quality of life is directly related to institutional support and adequate working conditions. Thus, burnout stems not only from high demands but also from an organizational model that weakens psychosocial protection, requiring concrete institutional measures that go beyond individual-level responsibility^(21,47,48)^.

Strategies to prevent burnout should prioritize women PMs and those with longer years of service, with emphasis on work reorganization, effective institutional support, and the promotion of psychosocially healthy environments. This context points to the need for structural changes in public safety, including greater recognition of these professionals and a reconfiguration of operational roles. It also calls for an intersectional approach that takes into account race/ethnicity, rank, symbolic exposure, and mechanisms of institutional silencing in analyses of PMs’ mental health^(6,49)^. Mindfulness-based interventions also appear promising for promoting mental health and underscore the importance of institutional investment in culturally sensitive support measures to address stress.

### Study limitations

The cross-sectional design precludes causal inference and allows only associations to be identified. The sample was recruited through convenience sampling, and the use of a self-report instrument may have introduced response bias, including social desirability bias, especially in a population that may be reluctant to acknowledge signs of psychological distress.

### Contributions to the field

This study contributes to nursing in the fields of occupational health and public health. Identifying burnout among PMs broadens understanding of psychological distress in high-risk occupational settings and supports efforts to promote mental health, prevent adverse outcomes, and implement psychosocial interventions. These findings can inform occupational health surveillance strategies and foster comprehensive, humanized care for public safety professionals.

## CONCLUSIONS

The findings reaffirm that burnout among Brazilian PMs reflects the mismatch between high job demands and limited institutional resources described by the Job Demands-Resources model. Exhaustion, mental distance, cognitive impairment, and emotional impairment capture the human cost of work overload, hierarchical rigidity, and the invisibility of psychological distress. Burnout thus emerges as a collective phenomenon, pointing to organizational weaknesses and to a management model that continues to normalize occupational wear and tear. Addressing this scenario requires institutional and intersectoral policies that expand protective resources and promote psychosocially healthy work environments. Alignment with SDGs 3 and 8 places these recommendations within a broader agenda of well-being, decent work, and safer, more equitable societies. In this context, nursing stands out as a strategic actor in public safety surveillance and policy development.

## Data Availability

The research data are available within the article.
