# Supplementary material for: Between the uniform and the toll: burnout and mental health among Brazilian Military Police
Source: Rev Bras Enferm. 2026 Sep 28;79:e20250429. doi: 10.1590/0034-7167-2025-0429 (PMC13623385; doi:10.1590/0034-7167-2025-0429)
Supplement: Translation

# Translation: Entre a farda e o desgaste: estudo sobre *burnout* e saúde mental na polícia brasileira

**DOI:** 10.1590/0034-7167-2025-0429pt

**Authors:** Caroline Schacker Evangelista, Kaihara de Freitas Furtado, Vitória Silva da Rosa, Ana Cristina Wesner Viana, Elisabete Maria das Neves Borges, Adriana Aparecida Paz

**Affiliations:** I Universidade Federal de Ciências da Saúde de Porto Alegre. Porto Alegre, Rio Grande do Sul, Brasil; II Hospital de Clínicas de Porto Alegre. Porto Alegre, Rio Grande do Sul, Brasil; III Escola Superior de Enfermagem do Porto. Porto, Portugal

**Keywords:** Burnout, Estresse Ocupacional, Polícia, Tecnologias em Saúde, Saúde Ocupacional.

## Abstract

**Objetivos:**

analisar a associação entre *burnout* e os fatores sociodemográficos, laborais e de saúde de policiais militares brasileiros.

**Métodos:**

estudo transversal realizado com 1.028 participantes entre novembro e dezembro de 2024. O *burnout* foi avaliado pelo *Burnout Assessment Tool*, com análises descritivas e inferenciais.

**Resultados:**

em 75,4% dos participantes, os níveis de *burnout* variaram de moderados a muito altos. Idade, tempo de atuação e sedentarismo apresentaram correlação negativa com *burnout*. Médias significativamente mais elevadas foram observadas entre mulheres, pessoas brancas, participantes com nível superior, turno fixo, sedentarismo, uso contínuo de medicação, histórico de atendimento psicológico e presença de vício.

**Conclusões:**

os achados evidenciam desequilíbrio entre altas demandas ocupacionais e escassez de recursos institucionais, em consonância com o modelo *Job Demands-Resources*. Destaca-se a urgência de políticas interdisciplinares, ambientes saudáveis e o cuidado com a saúde mental, alinhados aos Objetivos de Desenvolvimento Sustentável 3 e 8, com protagonismo da enfermagem.

## INTRODUÇÃO

Os transtornos mentais relacionados ao trabalho (TMRT) representam atualmente uma das principais causas de adoecimento de profissionais em contextos organizacionais complexos, desafiando a saúde pública e a segurança do trabalho no século XXI^(1)^. Destaca-se a síndrome de burnout (SB), caracterizada por exaustão emocional, distanciamento mental e redução das capacidades cognitivas e emocionais resultantes da exposição crônica ao estresse ocupacional^(2)^.

Desde sua inclusão na Classificação Internacional de Doenças (CID-11) pela Organização Mundial da Saúde (OMS), em 2022, a SB passou a ser oficialmente reconhecida como um fenômeno ocupacional, o que sublinha a necessidade de estratégias institucionais voltadas à vigilância, prevenção e cuidado em saúde mental no trabalho^(3)^. Apesar dos avanços conceituais, ainda são escassos os estudos aplicados que investigam a SB em profissões altamente expostas a riscos psicossociais, como é o caso dos profissionais da segurança pública.

Os policiais militares (PM) constituem um grupo emblemático entre as ocupações de maior vulnerabilidade psicossocial. Esses profissionais são expostos cotidianamente a condições adversas tais como jornadas de trabalho irregulares, violência frequente, escassez de recursos e tensões organizacionais, o que afeta não apenas o bem-estar individual, mas também o desempenho institucional e, consequentemente, a segurança da população^(4-6)^. O adoecimento dos PMs repercute ainda na estabilidade social, na qualidade dos serviços públicos e na confiança coletiva nas instituições de segurança^(7)^.

Para compreender esse cenário, é necessário adotar marcos teóricos capazes de articular as múltiplas dimensões do trabalho. Nesse contexto, destaca-se a Teoria das Demandas e Recursos do Trabalho (*Job Demands-Resources* - JD-R), proposta por Demerouti e colaboradores^(8)^ e posteriormente ampliada por Bakker e Demerouti^(9)^. De acordo com esse modelo, a SB resulta do desequilíbrio entre demandas laborais (emocionais, físicas e cognitivas) e os recursos disponíveis para o enfrentamento (p.ex., apoio institucional, autonomia e reconhecimento)^(10)^. Essa abordagem possibilita analisar de forma integrada a interação entre fatores individuais e organizacionais envolvidos no sofrimento ocupacional.

Com base nesse referencial, o *Burnout Assessment Tool* (BAT) foi desenvolvido como alternativa mais abrangente ao clássico *Maslach Burnout Inventory* (MBI). O BAT organiza os componentes da SB em quatro domínios principais (Exaustão, Distanciamento mental, Declínio cognitivo e Declínio emocional) e dois domínios secundários (Queixas psicológicas e Queixas psicossomáticas), permitindo uma leitura mais integrada do impacto psicossocial do trabalho sobre os indivíduos^(2,11)^. Além disso, o BAT ancora-se em um enquadramento teórico contemporâneo, ao incorporar distanciamento mental, declínio no controle cognitivo e declínio no controle emocional como componentes da SB, superando limitações do MBI^(12)^.

Neste estudo, optou-se pela utilização do BAT-33, na versão relacionada ao trabalho (*work related version*), em razão de sua aderência ao contexto ocupacional e de sua capacidade de mensurar sintomas vinculados às exigências e às condições laborais^(2,11)^. A adoção desse instrumento justifica se pela necessidade de produzir evidências que subsidiem políticas públicas e institucionais, reconhecendo a saúde mental como componente indissociável da saúde ocupacional e fortalecendo ações de prevenção, cuidado integral e valorização do trabalho do PM. Como diferencial, destaca-se o uso do BAT-33, instrumento mais abrangente e teoricamente consistente do que o tradicional MBI, por abarcar dimensões cognitivas e emocionais ainda pouco exploradas na literatura nacional. Ao identificar grupos vulneráveis e fatores associados à SB entre PMs brasileiros, o estudo amplia o debate científico e contribui, de forma inédita, para a formulação de práticas de enfrentamento baseadas em evidências.

## OBJETIVOS

Analisar a associação entre a síndrome de *burnout* e os fatores sociodemográficos, laborais e de saúde de policiais militares brasileiros.

## MÉTODOS

### Aspectos éticos

O estudo foi conduzido conforme diretrizes éticas nacionais e internacionais e aprovado pelo Comitê de Ética em Pesquisa da Universidade Federal de Ciências da Saúde de Porto Alegre (UFCSPA). Observou-se integralmente a Resolução nº 466/2012 do Conselho Nacional de Saúde^(13)^, que regulamenta pesquisas envolvendo seres humanos. Também foram seguidas as recomendações específicas para pesquisas em ambiente virtual e os princípios de privacidade e proteção de dados pessoais previstos na Lei nº 13.709/2018 (Lei Geral de Proteção de Dados - LGPD)^(14)^.

### Desenho, período e local do estudo

Trata-se de um estudo transversal, de abordagem quantitativa, descritiva e analítica. O presente artigo seguiu as diretrizes do *Strengthening the reporting of observational studies in epidemiology* (STROBE). O estudo foi realizado no ambiente virtual de divulgação científica do Grupo de Pesquisa em Tecnologia, Gestão, Educação e Segurança no Trabalho (TeGEST), vinculado à UFCSPA e registrado no Diretório de Grupos de Pesquisa do Conselho Nacional de Desenvolvimento Científico e Tecnológico (DGP-CNPq). Como estratégia de recrutamento, utilizou-se a página do grupo no Facebook^®^ (https://www.facebook.com/TeGEST.UFCSPA), ativa desde janeiro de 2019, e o perfil oficial do grupo no Instagram^®^ (https://instagram.com/tegest.ufcspa). A publicação do convite foi autorizada pelo vice-líder do grupo, mediante assinatura do termo de anuência.

### População e critérios de inclusão e exclusão

A população do estudo foi composta por 1.574 PMs brasileiros. A amostra foi obtida por conveniência, utilizando a técnica “bola de neve”, iniciada por meio de convites divulgados nas redes sociais do TeGEST com estímulo à partilha entre colegas da categoria. Adicionalmente, mensagens eletrônicas foram enviadas às Secretarias de Segurança Pública, Comandos Gerais, Casas Militares e associações de oficiais e praças de todos os estados do Brasil, solicitando apoio na divulgação da pesquisa aos efetivos nas corporações militares. Os critérios de inclusão foram: ser PM de carreira, estar em serviço ativo e possuir estabilidade funcional. Foram excluídos PMs aposentados, em afastamento temporário superior a 30 dias consecutivos (por motivos como licença-saúde, férias ou licenças similares), contratados temporariamente ou em período de estágio probatório. Após a aplicação dos critérios de elegibilidade e verificação da completude das respostas, obteve-se a amostra final composta por 1.028 PMs oriundos de diversas unidades federativas.

### Protocolo do estudo

O questionário foi disponibilizado por meio eletrônico (*https://bit.ly/PMQuestBR*) com QR Code. O formulário apresentava os critérios de elegibilidade e o Registro de Consentimento Livre e Esclarecido (RCLE), com opção para download do documento completo. A participação foi confirmada mediante a aceitação do RCLE, registrada no próprio formulário.

A coleta de dados ocorreu entre novembro e dezembro de 2024, por meio de um questionário on-line estruturado em duas seções: (1) *Burnout*, avaliado pelo BAT-33 (*work-related version*); (2) Caracterização sociodemográfica, laboral e de saúde dos PMs. O BAT foi traduzido e adaptado para o contexto brasileiro, com evidências de validade e confiabilidade descritas em amostra brasileira de trabalhadores (n = 2.217), de múltiplos grupos ocupacionais, no âmbito de estudo transcultural que também incluiu trabalhadores portugueses (n = 886). O instrumento é composto por 33 itens distribuídos em quatro domínios principais (Exaustão, Distanciamento mental, Declínio no controle cognitivo e Declínio no controle emocional) e dois domínios secundários (Queixas psicológicas e Queixas psicossomáticas)(2). A adoção da versão *work-related* justifica-se por sua adequação ao contexto ocupacional, permitindo avaliar o impacto das demandas e do ambiente de trabalho sobre a saúde mental. Além disso, sua utilização tem sido recomendada em categorias profissionais expostas a elevada demanda e estresse, o que respalda sua aplicabilidade ao público investigado^(2,15)^.

### Análise dos resultados e estatística

Os dados foram armazenados na plataforma Google Drive^®^, na planilha do Google Sheets^®^, e exportados em formato Excel^®^ da Microsoft^®^ para um computador local, assegurando o anonimato e a proteção dos dados sensíveis. As variáveis foram codificadas conforme um manual de codificação específico para garantir consistência e padronização de algumas respostas. Em seguida, os dados foram importados para o *software Statistical Package for the Social Sciences* ^®^ para análise descritiva e inferencial. As variáveis foram apresentadas em frequências absolutas e relativas, medidas de tendência central, dispersão e percentis, de acordo com a natureza e distribuição dos dados. A normalidade foi verificada pelo teste de Kolmogorov-Smirnov (p < 0,05). Para a análise inferencial, adotou-se a Correlação de Pearson e o teste *t* de Student, considerando nível de significância de 5%.

## RESULTADOS

Participaram do estudo 1.028 PMs, oriundos de 225 municípios, distribuídos em 20 unidades federativas do Brasil. A Região Norte apresentou o maior número de participantes (491; 47,8%), seguida das Regiões Sul (284; 27,6%), Centro-Oeste (134; 13,0%), Nordeste (111; 10,8%), Sudeste (7; 0,7%), e um participante não informou a região de origem (1; 0,1%). A amostra foi composta majoritariamente pelo sexo masculino (842; 81,9%) e por heterossexuais (996; 96,9%), com idade média de 41,9±6,5 anos (mínima de 23 e máxima de 60 anos.) Quanto à raça/cor, autodeclararam-se pardos (510; 49,6%), brancos (387; 37,6%), negros (118; 11,5%), amarelos (5; 0,5%) e indígenas (4; 0,4%); e 4 (0,4%) não informaram. Em relação à situação conjugal, 807 (78,5%) eram casados ou viviam em união estável. A escolaridade predominante foi o ensino nível superior (817; 79,5%), seguido do ensino médio (177; 17,2%), curso técnico-profissionalizante (24; 2,3%), ensino fundamental (8; 0,8%); e 2 (0,2%) não informaram. A Tabela 1 apresenta as características laborais e de saúde dos PMs.

**Tabela 1:** Características laborais e de saúde dos policiais militares, Brasil, 2025

Características laborais e de saúde	n (%)
**Posto/Graduação**	
Sargento	438 (42,5)
Cabo	158 (15,4)
Soldado	127 (12,3)
Tenente	85 (8,3)
Major	58 (5,6)
Tenente-coronel	53 (5,2)
Subtenente	52 (5,1)
Capitão	39 (3,8)
Coronel	15 (1,5)
Não informou	3 (0,3)
**Tempo de atuação (em anos)**	17,6 (7,7)^*^
**Função**	
Administração	450 (43,8)
Policiamento	416 (40,5)
Administração e policiamento	48 (4,7)
Outras funções	90 (8,7)
Não informou	24 (2,3)
**Turno de trabalho**	
Alternado	492 (47,9)
Manhã e tarde	314 (30,4)
Tarde	124 (12,1)
Manhã	84 (8,2)
Noite	12 (1,2)
Não informou	2 (0,2)
**Carga horária semanal (horas)**	43,8 (9,9)^*^
**Unidade federativa**	
Pará	307 (29,8)
Santa Catarina	161 (15,7)
Rio Grande do Sul	123 (11,9)
Goiás	109 (10,6)
Bahia	79 (7,7)
Tocantins	74 (7,2)
Rondônia	60 (5,8)
Acre	24 (2,3)
Alagoas	20 (1,9)
Roraima	15 (1,5)
Mato Grosso	13 (1,3)
Amazonas	11 (1,1)
Distrito Federal	8 (0,8)
Rio de Janeiro	6 (0,6)
Sergipe	6 (0,6)
Maranhão	4 (0,4)
Mato Grosso do Sul	4 (0,4)
São Paulo	1 (0,1)
Pernambuco	1 (0,1)
Rio Grande do Norte	1 (0,1)
Não informou	1 (0,1)
**Atividade física (sim)**	849 (82,6)
Frequência de atividade física (dias)	3,3 (1,4)^*^
**Medicação de uso contínuo (sim)**	332 (32,3)
**Atendimento psicológico (sim)**	452 (44)
Atendimento realizado na corporação	185 (40,9)
Prefiro não dizer	68 (6,5)
**Algum vício (sim)**	212 (20,6)
Substâncias consumidas	1,2 (0,5)^*^

* *Média ± Desvio-padrão.*

A maioria dos PMs (860; 83,6%) ocupava os postos de soldado, cabo, sargento e de oficial subalterno, como subtenente e tenente. O tempo de serviço variou de 1 a 40 anos de atuação, com predominância de PMs com até 19 anos na instituição, e 534 (51,9%) eram vinculados a turnos fixos. Quanto a “Outras funções”, desempenhadas por 90 participantes, 23 (25,5%) atuavam no quadro de saúde; 20 (22,3%) na Casa Militar; 12 (13,3%) na Defesa Civil; 9 (10,1%) como Agentes de Inteligência; 8 (8,9%) no Serviço de Guarda; 6 (6,7%) em unidades educacionais; 3 (3,3%) na Central de Emergências; 3 (3,3%) na Cavalaria; 2 (2,2%) como Armeiros, 2 (2,2%) na Unidade Artística e 2 (2,2%) em Operações Especiais. Sobre os vícios, apenas 149 (70,2%) informaram o tipo de substância consumida, resultando em 192 respostas, obtendo um média de 1,2±0,5 substâncias utilizadas. A prevalência de consumo foi de álcool (87; 45,3%), seguido de tabaco (38; 19,8%), compulsão alimentar (26; 13,5%), medicamentos sem prescrição (16; 8,3%) e maconha (1; 0,5%). Destaca-se que 24 (12,6%) participantes optaram por registrar outros vícios, embora houvesse como opções de resposta: maconha, cocaína, ecstasy e crack. Outros 63 (29,8%) participantes que declararam possuir vício preferiram não informar ou não responderam sobre o tipo de vício. A prevalência de sintomas da SB é apresentada na Tabela 2.

**Tabela 2:** Prevalência de sintomas da síndrome de *burnout* dos policiais militares, Brasil, 2025

Dimensões e domínios	Média (DP^*^)	Baixo n (%)	Moderado n (%)	Alto n (%)	Muito alto n (%)	IC 95% α Cronbach
**BAT-33**	2,2 (0,7)	253 (24,6)	516 (50,2)	206 (20)	53 (5,2)	1,65-3,14 0,968
**Sintomas primários**	2,2 (0,7)	268 (26,1)	506 (49,2)	202 (19,6)	52 (5,1)	1,65-2,90 0,958
Exaustão	2,6 (0,8)	253 (24,6)	504(49)	228 (22,2)	43 (4,2)	2,22-2,90 0,930
Distanciamento mental	1,9 (0,9)	308 (30)	464 (45,1)	207 (20,1)	49 (4,8)	1,70-2,44 0,873
Declínio no controle cognitivo	2,1 (0,8)	267 (26)	517 (50,2)	200 (19,5)	44 (4,3)	1,91-2,54 0,886
Declínio no controle emocional	1,8 (0,7)	329 (32)	450 (43,7)	198 (19,3)	51 (5,0)	1,65-2,18 0,904
**Sintomas secundários**	2,4 (0,8)	292 (28,4)	499 (48,5)	187 (18,2)	50 (4,9)	1,77-3,14 0,921

* *DP - Desvio-padrão.*

A idade apresentou correlação negativa, fraca e estatisticamente significativa com todas as dimensões e domínios do BAT-33, indicando discreta redução dos escores de SB com o aumento da idade: BAT-33 (r = -0,114; p < 0,001), sintomas primários (r = -0,111; p < 0,001), Exaustão (r = -0,128; p < 0,001), Distanciamento mental (r = -0,070; p = 0,024), Declínio no controle cognitivo (r = -0,100; p = 0,001), Declínio no controle emocional (r = -0,071; p = 0,022) e sintomas secundários (r = -0,105; p = 0,001). O tempo de atuação como PM correlacionou-se negativamente com Exaustão (r = -0,085; p = 0,007), sugerindo menor desgaste entre os mais experientes. Não foram observadas associações significativas entre carga horária semanal e os escores do BAT-33 (p > 0,05).

A frequência semanal de atividade física apresentou correlações negativas e significativas com todas as dimensões e domínios do BAT-33 (r = -0,149; p < 0,001), sintomas primários (r = -0,138; p < 0,001), Exaustão (r = -0,145; p < 0,001), Distanciamento mental (r = -0,088; p = 0,009), Declínio no controle cognitivo (r = -0,118; p < 0,001), Declínio no controle emocional (r = -0,127; p < 0,001) e sintomas secundários (r = -0,151; p < 0,001). Em contrapartida, o número de substâncias consumidas por vício correlacionou-se positivamente com todas as dimensões e domínios do BAT-33 (r = 0,325; p < 0,001), sintomas primários (r = 0,316; p < 0,001), Exaustão (r = 0,319; p < 0,001), Distanciamento mental (r = 0,240; p = 0,003), Declínio no controle cognitivo (r = 0,224; p = 0,006), Declínio no controle emocional (r = 0,305; p < 0,001) e sintomas secundários (r = 0,308; p < 0,001). A Tabela 3 apresenta as associações entre as dimensões e domínios do BAT-33 e as variáveis sociodemográficas, laborais e de saúde dos PMs.

**Tabela 3:** Associação da síndrome de *burnout* e características sociodemográficas, laborais e de saúde dos policiais militares, Brasil, 2025

Variáveis	BAT-33		Sintomas primários		Exaustão		Distanciamento mental		Declínio no controle cognitivo		Declínio no controle emocional		Sintomas secundários	
	Média (DP^*^)	Valor de *p* ^†^	Média (DP^*^)	Valor de *p* ^†^	Média (DP^*^)	Valor de *p* ^†^	Média (DP^*^)	Valor de *p* ^†^	Média (DP^*^)	Valor de *p* ^†^	Média (DP^*^)	Valor de *p* ^†^	Média (DP^*^)	Valor de *p* ^†^
**Sexo**														
Masculino	2,2(0,7)	**0,008**	2,1(0,7)	0,097	2,6(0,8)	0,238	1,9(0,9)	0,925	2,1(0,8)	**0,001**	1,8(0,7)	0,105	2,3(0,8)	**<0,001**
Feminino	2,4(0,7)		2,2(0,6)		2,7(0,7)		1,9(0,8)		2,3(0,8)		1,9(0,8)		2,6(0,8)	
**Raça/Cor**														
Pardo, negro, indígena e amarelo	2,1(0,7)	**< 0,001**	2,1(0,7)	**< 0,001**	2,5(0,8)	**0,014**	1,8(0,8)	**< 0,001**	2,0(0,8)	**< 0,001**	1,7(0,7)	**< 0,001**	2,3(0,8)	**< 0,001**
Branco	2,4(0,7)		2,3(0,7)		2,7(,0,8)		2,0(0,9)		2,3(0,8)		1,9(0,8)		2,5(0,8)	
**Formação**														
Ensino médio	2,1(0,7)	**< 0,001**	2,0(0,7)	**< 0,001**	2,4(0,8)	**0,002**	1,8(0,8)	0,058	1,9(0,8)	**< 0,001**	1,7(0,7)	**0,002**	2,2(0,8)	**< 0,001**
Ensino superior	2,3(0,7)		2,2(0,7)		2,6(0,8)		1,9(0,9)		2,2(0,8)		1,8(0,8)		2,4(0,8)	
**Posto/graduação**														
Baixo escalão	2,2(0,7)	0,221	2,2(0,7)	0,226	2,6(0,8)	0,228	2,0(0,9)	**0,002**	2,1(0,9)	0,371	1,8(0,8)	0,535	2,4(0,9)	0,278
Alto escalão	2,2(0,6)		2,1(0,6)		2,5(0,7)		1,8(0,8)		2,1(0,7)		1,8(0,7)		2,3(0,7)	
**Tempo de atuação**														
≤ 19 anos	2,2(0,7)	0,277	2,2(0,7)	0,270	2,6(0,8)	**0,014**	1,9(0,9)	0,540	2,1(0,8)	0,833	1,8(0,7)	0,918	2,4(0,8)	0,355
≥ 20 anos	2,2(0,7)		2,1(0,7)		2,5(0,8)		1,9(0,9)		2,1(0,8)		1,8(0,7)		2,3(0,8)	
**Turno de trabalho**														
Fixo	2,3(0,7)	0,159	2,2(0,7)	0,264	2,6(0,8)	0,748	1,9(0,8)	0,509	2,2(0,8)	**< 0,001**	1,8(0,7)	0,235	2,4(0,8)	0,070
Alternado	2,2(0,7)		2,1(0,7)		2,6(0,8)		1,9(0,9)		2,0(0,8)		1,8(0,8)		2,3(0,8)	
**Atividade física**														
Não	2,4(0,7)	**< 0,001**	2,3(0,7)	**0,001**	2,8(0,8)	**< 0,001**	2,1(0,9)	**0,002**	2,3(0,8)	**0,004**	2,0(0,8)	**0,009**	2,7(0,8)	**< 0,001**
Sim	2,2(0,7)		2,1(0,7)		2,6(0,8)		1,9(0,9)		2,1(0,8)		1,8(0,7)		2,3(0,8)	
**Uso contínuo de medicamento**														
Não	2,1(0,6)	**< 0,001**	2,0(0,6)	**< 0,001**	2,5(0,8)	**< 0,001**	1,8(0,8)	**< 0,001**	2,0(0,7)	**< 0,001**	1,7(0,7)	**< 0,001**	2,2(0,7)	**< 0,001**
Sim	2,5(0,7)		2,4(0,7)		2,8(0,8)		2,1(0,9)		2,4(0,9)		2,0(0,8)		2,7(0,8)	
**Atendimento psicológico**														
Não	1,9(0,5)	**< 0,001**	1,9(0,5)	**< 0,001**	1,9(0,5)	**< 0,001**	1,6(0,7)	**< 0,001**	1,8(0,7)	**< 0,001**	1,5(0,5)	**< 0,001**	2,0(0,6)	**< 0,001**
Sim	2,6(0,7)		2,5(0,7)		2,5(0,8)		2,2(0,9)		2,5(0,8)		2,1(0,8)		2,8(0,8)	
**Vício**														
Não	2,2(0,7)	**< 0,001**	2,1(0,7)	**< 0,001**	2,5(0,8)	**< 0,001**	1,8(0,8)	**< 0,001**	2,0(0,8)	**< 0,001**	1,7(0,7)	**< 0,001**	2,3(0,8)	**< 0,001**
Sim	2,5(0,8)		2,4(0,8)		2,8(0,8)		2,2(1,0)		2,4(0,9)		2,1(0,9)		2,6(0,9)	

* *Desvio-padrão; †nível de significância (p < 0,05).*

A análise dos fatores associados à SB entre PMs evidenciou maior vulnerabilidade entre profissionais do sexo feminino, que apresentaram médias mais elevadas no BAT-33, declínio no controle cognitivo e sintomas secundários. Policiais brancos também obtiveram escores mais altos em todos os domínios e dimensões, sugerindo possíveis influências socioculturais e ocupacionais. Profissionais com ensino superior apresentaram maiores médias na maioria das dimensões, exceto em distanciamento mental, o que pode indicar maior percepção ou exposição a estressores laborais.

O posto/graduação demonstrou associação significativa com distanciamento mental, com destaque para os praças (soldados, cabos, sargentos, subtenentes e tenentes), possivelmente em razão da sobrecarga e da menor autonomia. A exaustão emocional foi mais acentuada entre PMs com até 19 anos de atuação, sugerindo maior desgaste em fases intermediárias da carreira. Turnos fixos associaram-se ao declínio no controle cognitivo, indicando efeitos negativos da rigidez na jornada sobre a saúde ocupacional.

A prática regular de atividade física foi um fator protetor, com menores escores em todas as dimensões e domínios do BAT-33. Em contrapartida, o uso contínuo de medicamento relacionou-se a maiores médias, possivelmente refletindo comorbidades ou estratégias de enfrentamento menos eficazes. Policiais em acompanhamento psicológico também apresentaram escores mais elevados, sugerindo maior nível de estresse. O uso de substâncias esteve associado a maior comprometimento psicossocial, evidenciando comportamentos de risco relacionados à SB. Não foram observadas associações estatisticamente significativas entre as dimensões e domínios do BAT-33 e as variáveis “idade superior a 40 anos”, “situação conjugal” e “disponibilidade de atendimento psicológico na corporação” (p > 0,05), indicando ausência de influência direta sobre os níveis da SB.

## DISCUSSÃO

A interpretação dos resultados dialoga com o modelo teórico JD-R, que concebe a SB como produto do desequilíbrio entre demandas ocupacionais elevadas e escassez de recursos institucionais e pessoais. No contexto policial, essas demandas incluem exposição ao risco, pressão hierárquica, longas jornadas e carga emocional elevada, enquanto os recursos de apoio social, reconhecimento e autonomia são limitados. O BAT revela os efeitos desse desequilíbrio em dimensões mensuráveis, como Exaustão, Distanciamento mental, Declínio no controle cognitivo e Declínio no controle emocional, o que permite compreender a SB como fenômeno tanto individual quanto estrutural e ratifica a necessidade de políticas institucionais que fortaleçam os recursos de enfrentamento e proteção à saúde mental dos PMs^(16,17)^.

O BAT apresenta avanços conceituais sobre o MBI ao integrar, como domínios principais, o Distanciamento mental, o Declínio no controle cognitivo e o Declínio no controle emocional, ampliando a identificação de efeitos do trabalho sobre a saúde mental e reconhecendo a complexidade psicossocial do sofrimento ocupacional^(2,10,18)^. Nesse escopo, o declínio no controle cognitivo, manifestado por lapsos de memória e lentidão no processamento de informações, evidencia a importância de intervenções preventivas e estratégicas para a preservação da capacidade operacional dos PMs^(5,19-22)^. Além do mais, redefine o cinismo como distanciamento mental, entendido como um processo de afastamento cognitivo e afetivo progressivo, associado à perda de sentido e de engajamento profissional^(2,15,18)^. Por fim, ao avaliar sintomas secundários, como distúrbios do sono, taquicardia, cefaleia, dores musculares e desconfortos gastrointestinais, o instrumento incorpora manifestações que constituem reflexos do estresse crônico, frequentemente naturalizados no cotidiano policial^(2,15,18)^.

A amostra, majoritariamente masculina e com média etária superior a 40 anos, reflete o envelhecimento da força de trabalho em uma estrutura hierárquica rígida. As mulheres apresentam maior vulnerabilidade à SB, especialmente no tocante à exaustão emocional, intensificada por ambientes masculinizados que invisibilizam o sofrimento laboral^(5,23,24)^. A idade e o tempo de serviço configuram fatores de risco, associados ao desgaste acumulado e à exposição prolongada ao estresse^(25)^. A maioria atua como praças, em turnos alternados e com acúmulo de funções, situação que eleva o risco de exaustão emocional e distanciamento mental^(6,26)^. A insatisfação com a estrutura hierárquica e a sobrecarga imposta aos postos inferiores apontam que o adoecimento não decorre apenas de fatores individuais, mas da escassez de recursos de enfrentamento oferecidos pelo modelo organizacional.

Ter ensino superior não se traduz necessariamente em ascensão funcional, gerando frustração e baixa realização profissional^(27)^. O reconhecimento por superiores influencia a saúde mental positivamente, enquanto sua ausência favorece estresse ocupacional^(4,27-29)^. Os PMs brancos apresentaram maiores escores de SB, possivelmente em função de maior visibilidade, cobrança institucional e autocobrança, enquanto PM negros podem relatar menos sofrimento por naturalização da invisibilidade emocional ou desconfiança institucional na preservação da confidencialidade^(30-32)^.

Os sintomas intensos de SB estão associados à intenção de desligamento precoce da carreira, sobrecarga funcional e baixo suporte organizacional, evidenciando risco à permanência profissional^(33)^. Pesquisa semelhante realizada com policiais na Tailândia identificou que a sobrecarga funcional, somada à rigidez hierárquica e à falta de reconhecimento, tem intensificado a exaustão psíquica e o desejo de afastamento da carreira^(34)^. Em contextos de altas exigências e baixo retorno subjetivo, a SB gera o adoecimento e desmotivação, comprometendo a saúde mental e a permanência na profissão^(16,17)^.

A exposição contínua à dor, à violência e à vulnerabilidade leva ao esgotamento emocional e exaustão afetiva, o que favorece o padrão de distanciamento mental dos PMs identificado como estratégia de autopreservação^(35)^. Esse distanciamento mental, frequentemente manifestado como indiferença ou cinismo, não indica necessariamente desprezo, mas um modo de lidar com o estresse crônico^(2,11,15,18)^.

Apesar de muitos PMs relatarem boa saúde, os dados revelam consumo de medicamentos, busca por atendimento psicológico e relatos de vícios, sugerindo dissonância entre percepção e realidade do adoecimento psíquico. Em contextos militares, cujos valores predominantes são de resistência emocional, a admissão de fragilidade é interpretada como fraqueza, o que contribui para a negação institucional do adoecimento e retarda o acesso a cuidados adequados^(36)^. Estudos revelam que, na ausência de espaços seguros para expressão emocional, muitos PMs recorrem a estratégias individuais como automedicação, uso de substâncias e isolamento, as quais, embora paliativas, tendem a agravar o quadro de esgotamento e distanciamento psíquico^(37,38)^.

Entretanto, esses dados não indicam, necessariamente, a existência de políticas institucionais de cuidado. Ao contrário, podem evidenciar a ausência de respostas estruturais e a individualização da responsabilidade pela saúde mental^(39)^. Um estudo aponta que, mesmo diante da alta prevalência de transtornos mentais, a maioria dos PMs recorre a cuidados autônomos, sem o suporte institucional adequado^(40)^. Barreiras como o estigma e a desconfiança nos serviços da própria corporação limitam a busca por ajuda, enquanto o acúmulo de estressores cotidianos agrava a vulnerabilidade psíquica^(41)^. Esse cenário realça a necessidade de intervenções sistemáticas e contínuas que transcendam a responsabilização individual.

A saúde mental ainda é tratada como responsabilidade individual, com escassez de respostas institucionais, estigma e desconfiança da corporação, limitando o acesso a cuidados adequados e o engajamento em práticas de autocuidado diante da cultura da invulnerabilidade^(5,35)^. A ausência de apoio institucional leva o PM a evitar a busca por ajuda, agravando sintomas de estresse, exaustão e risco de transtornos como o transtorno de estresse pós-traumático (TEPT)^(42)^. Por outro lado, as intervenções estruturadas baseadas em *mindfulness* têm-se mostrado promissoras no desenvolvimento da atenção plena, regulação emocional e redução da SB, sobretudo quando inseridas em contextos hierárquicos com suporte organizacional^(43-45)^.

A participação de PMs de diferentes regiões (com forte representação do Norte e Sul), associada ao tamanho amostral do estudo, confere maior robustez aos achados e sugere que a SB entre PMs constitui um problema sistêmico. A exposição recorrente à violência, baixa capacidade de trabalho e consumo de álcool associaram-se à piora da saúde mental com maior risco de esgotamento, confirmando achados convergentes na literatura nacional, que aponta a sobrecarga funcional e os comportamentos de risco como elementos centrais no adoecimento da tropa^(36,46)^. A percepção de qualidade de vida relaciona-se diretamente com o suporte institucional e condições de trabalho adequadas. Assim, a SB não decorre apenas de alta demanda, mas também de um modelo organizacional que fragiliza a proteção psicossocial, requerendo medidas institucionais concretas que superem a responsabilização individual^(21,47,48)^.

As estratégias de prevenção à SB devem priorizar PMs mulheres e com maior tempo de serviço, enfatizando a reorganização do trabalho, o suporte institucional efetivo e a promoção de ambientes psicossocialmente saudáveis. Esse contexto aponta para a necessidade de transformações estruturais na segurança pública, com valorização dos profissionais e da reconfiguração das funções operacionais, além de exigir uma abordagem interseccional que considere raça/cor, posição funcional, exposição simbólica e mecanismos de silêncio institucional na análise da saúde mental dos PMs^(6,49)^. As intervenções baseadas em *mindfulness* destacam-se como promissoras para a promoção da saúde mental e ressaltam a importância de investimentos institucionais culturalmente sensíveis de acolhimento no enfretamento do estresse.

### Limitações do estudo

O delineamento transversal impossibilita a inferência de causalidade entre as variáveis, restringindo-se à identificação de associações. A amostra foi composta por conveniência, e o uso de instrumento de autorrelato pode ter introduzido vieses de resposta, como o de desejabilidade social, sobretudo em uma população potencialmente resistente a expressar sinais de adoecimento psíquico.

### Contribuições para a Área

Este estudo contribui para a enfermagem no campo da saúde do trabalhador e da saúde coletiva. A identificação da SB em PMs amplia a compreensão sobre o adoecimento psíquico em contextos de risco ocupacional elevado, subsidiando ações de promoção da saúde mental, prevenção de agravos e implementação de intervenções psicossociais. Tais ações podem orientar estratégias de vigilância em saúde do trabalhador, promovendo a integralidade e a humanização do cuidado aos profissionais da segurança pública.

## CONCLUSÕES

Os resultados deste estudo reafirmam que a SB entre PMs brasileiros reflete o descompasso entre demandas ocupacionais elevadas e recursos institucionais limitados, como preconiza o modelo JD-R. Exaustão, distanciamento mental, declínio no controle cognitivo e declínio no controle emocional traduzem o custo humano imposto por sobrecarga, rigidez hierárquica e invisibilidade do sofrimento psíquico. Trata-se de um fenômeno coletivo, indicador das fragilidades organizacionais e de um modelo de gestão que ainda naturaliza o desgaste laboral. Superar esse cenário exige políticas institucionais e intersetoriais que ampliem recursos de proteção e promovam ambientes psicossociais saudáveis. O alinhamento aos ODS 3 e 8 situa essas recomendações no marco de promoção do bem estar, do trabalho decente e de sociedades mais seguras e equitativas. Nesse contexto, a enfermagem se destaca como agente estratégica na vigilância e formulação de políticas em segurança pública.

## Data Availability

Os dados de pesquisa estão disponíveis no corpo do artigo.
